# Learning is a means to progress and empowerment for health systems

**DOI:** 10.1136/bmjgh-2022-010572

**Published:** 2022-09-20

**Authors:** Kabir Sheikh, David Peters, Irene Akua Agyepong, Seye Abimbola, Abdul Ghaffar, Soumya Swaminathan

**Affiliations:** 1 Alliance for Health Policy and Systems Research, World Health Organization, Geneve, Switzerland; 2 Bloomberg School of Public Health, Johns Hopkins University, Baltimore, Maryland, USA; 3 Dodowa Health Research Centre, Ghana Health Service, Accra, Greater Accra, Ghana; 4 School of Public Health, University of Sydney, Sydney, New South Wales, Australia; 5 Science Division, World Health Organization, Geneva, Switzerland

**Keywords:** Health systems, Health policy

There is widespread consensus that learning is crucial for the performance of health systems and the achievement of broader health goals. However, this consensus is not equally matched by shared knowledge and understanding of how health systems learn, or of how to improve health systems learning across different contexts. The very term ‘learning health systems’ (and variations of it) is not new—it has been invoked for more than a decade in different contexts, and with quite disparate connotations.^1^

In September 2021, the Alliance for Health Policy and Systems Research published its flagship report: ‘Learning health systems: pathways to progress’.^2^ This report, building on the body of existing theories and frameworks of learning organisations, was informed by experiential cases from 14 countries and guidance from an advisory group of country policy-makers and health system experts, and reflects a concerted attempt to develop the learning health systems concept.

In this editorial, we, the editors and members of the advisory group for the report, summarise some of these key advances and their wider significance. Together with the other articles in this special series on learning health systems, we are hopeful that the ideas in this editorial will serve as a useful guide for further thought, and for actions and investments in learning to strengthen health systems worldwide.

## Learning helps all functions of health systems at all levels

All too often, health systems have been taken to be synonymous with healthcare systems or health services. Some initiatives that use the terminology of learning health systems have focused on decision-making in healthcare settings, reflecting the conflation of ‘health systems’ with ‘healthcare systems’ or ‘health services’.^3–5^ This report, however, adopts a broader understanding of health systems.

The global health and development community has, for the past 30 years, advanced and applied an understanding of health systems that extends beyond healthcare services, to include multiple functions that provide mutual support for each other.^6–8^ Furthermore, the ‘health system’ is not synonymous with the health sector. A health system promotes, restores and maintains health, not only through clinical care and public health programmes but also through efforts to improve the social and structural determinants of health, many of which lie outside the health sector itself.^6–10^ A health system is most simply described as being made up of component parts (eg, stakeholders and organisations) and interactions (eg, functions and services) that promote, restore and maintain health and that, taken together, form a unified whole.^6^

**Figure 1:**
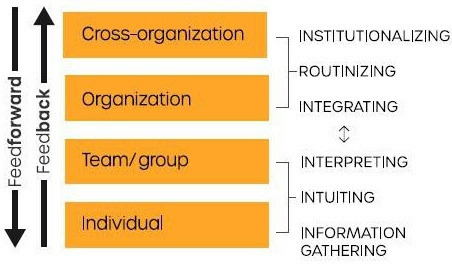
Learning occurs at all levels of a health system (adapted from Jenkin^2 16^).

Learning occurs at all levels of health systems and involves the generation, acquisition and sharing of knowledge and changing behaviour based on new insights (figure 1). Learning at the individual level entails information gathering from different sources, gaining tacit knowledge through experience and interpretation of these knowledge inputs. In contrast, team and group-level learning tends to involve the collective interpretation of knowledge through dialogue and exchange, and the development of shared understanding about issues, problems and solutions.^11^ Learning at organisation and cross-organisation levels takes place through the formalisation of rules and procedures that are conducive to learning. Ultimately, it is important that learning is integrated and institutionalised so that it can be shared and used on a regular basis to drive improvements throughout the system in a sustained manner.^12^

## Learning is more than information transfer: action and deliberation are as important

**Figure 2:**
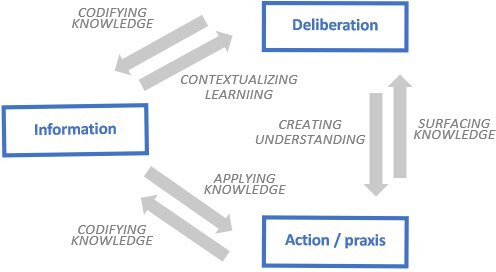
Means of learning in health systems.^2^

Information is gathered, processed and deployed to meet the diverse learning aims of health systems, including measuring success and failure, anticipating trends and discovering new approaches to address problems.^13 14^ Such information is found in explicit or codified form and may be spoken or written, saved, transmitted and downloaded remotely.^15 16^ However, learning in health systems results not only from the transmission of information but is also produced through acts of human deliberation, and through action and praxis (figure 2).^15 17 18^

Processes of dialogue and reflection are necessary to contextualise problems and advance collective understanding on how to anticipate, prevent and solve them.^11 15^ The collective understanding that is generated through such processes is more than an aggregate of individual knowledge because it is enriched by new knowledge and by insights produced through debate and dialogue.^19–21^ Within health systems, these processes encompass a range of non-peer and peer engagements—including stakeholder consultations, team meetings, research collaborations, conferences and community and public engagement fora—and may occur in-person or through technology-enabled platforms.^22–24^

A good example of deliberative learning is the National Health Assembly (NHA) in Thailand, which brings together government leaders, academics and representatives from other sectors, civil society, professional associations and community groups to foster dialogue on Universal Health Coverage (UHC) planning and implementation. The NHA has enhanced mutual understanding among stakeholders, even though the prioritisation of the outcomes of these deliberations in policy-making remains a challenge.^25^

A third means of learning in health systems is through action and praxis.^15 26^ People, whether individually or as part of a team, group or organisation, learn through the practice and iteration of tasks and projects.^27–30^ Complex social systems are repositories of such tacit and experiential learning, which is held by diverse actors within or across different parts of the system.^17^ Experiential learning gives rise to innovations and ‘good practices’, which can then be learnt by other actors in different parts of the system or in other systems. According to Stiglitz, this occurs through horizontal learning processes (eg, on-the-job mentoring, team learning, study tours, secondments), which entail ‘seeing how it is done’, ‘being shown how to do it’ or a combination of both.^15^

In Kenya, for example, nursing personnel who serve nomadic Somali communities engaged in participatory learning exercises, working with these communities over time to understand their health problems and practices, and their perceptions of healthcare services and information networks. The relationships that were built as a result enabled the nurses to provide more effective services attuned to the communities’ lifestyles.^31^

## Learning has diverse benefits for health systems: from correcting errors to greater self-reliance

**Figure 3:**
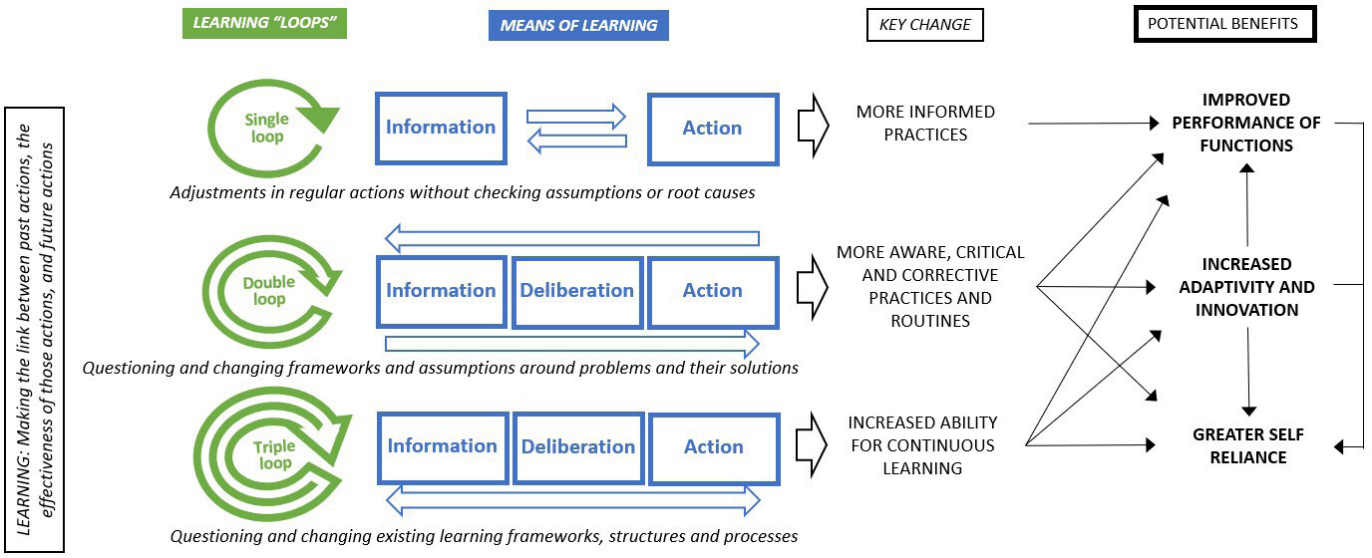
Diverse benefits of learning health systems.^2^

Learning has different types of benefits for health systems (figure 3). In its simplest ‘single-loop’ form, learning enables individuals, teams and organisations to adapt and improve their regular practices to perform their stated functions more effectively.^32^ Implementation research initiatives, for example, are reported to play a role in improving case findings, diagnosis and treatment to meet central programme targets, as part of several national tuberculosis programmes in the Asia Pacific region and are used increasingly to aid programme managers based in low-income and middle-income countries (LMICs) around the world to improve the delivery of programmes to prevent non-communicable diseases.^33–36^

However, deeper forms of learning can also help health systems to embrace change better. Such changes can range from evolving societal expectations, population characteristics and disease patterns to external shifts such as the shocks caused by political and economic upheavals, natural disasters or global ecological trends. Health systems are ready to go back to the drawing board and rethink policies and strategies (double-loop learning), are in a better position to innovate and adapt their actions to meet contextual changes. In Mexico, evidence on how catastrophic and impoverishing health spending was impacting citizens led leaders to question their assumptions about how health financing should be managed and what kinds of financial protection were needed. The resulting policy changes led to the creation of the Seguro Popular (Popular Health Insurance), which has since protected many households from impoverishing health expenditures.^37^

Some health systems in LMICs have not fully integrated learning frameworks and structures into their health systems operations—this reduces their ability to strategise, act, and optimise their use of existing resources, and can create dependencies on external actors for knowledge and intelligence. Such health systems are strengthened through triple-loop learning, which is about ‘learning how to learn’—a rethinking of the learning frameworks and methods used within health systems. An illustrative example of triple-loop learning comes from the Nigerian experience with Lassa fever helped evolve new learning processes (ie, outbreak surveillance software and after action reviews), enhancing the ability of the health system to learn while tackling epidemics, and also helping to shape the preparation for epidemics such as COVID-19 in the future.

## Conclusion

There have been incremental advances in thinking about health systems strengthening over the past 30 years—on what makes a strong health system, and on appropriate paradigms to inform efforts to strengthen systems worldwide. The WHO ‘health system building blocks’ framework was developed to communicate the kinds of inputs that are required to strengthen health systems to a wide audience.^38^ In more recent years, there has been a growing recognition that health systems are complex and adaptive social systems that adjust and transform in response to their environment.^39^ These concepts have also helped to position health systems as being people centred, and have breathed dynamic life into the building blocks. 40–44 Resilience has also re-emerged as a relevant concept to describe an essential characteristic of a strong health system.^45–47^ Resilience in health systems highlights the importance of governance, institutional arrangements, the use of information and the many connected functions in how health systems respond to stress and shocks.

Learning is a forward-looking and actionable lens through which to view the strengthening of health systems. It builds on existing frameworks of health systems strengthening, and is linked to the agendas of improved equity, efficiency, resilience, people centredness, self-reliance and improved quality. The importance of learning is increasingly pronounced in the current context, with the growing focus on the abilities of health systems to identify and respond to pandemics, to transition from foreign aid to domestic funds, and to capitalise on the information revolution to achieve their goals.^48 49^ Ultimately, learning is a route to progress and empowerment for health systems—particularly those in LMICs—by developing the inbuilt ability to generate and use the knowledge and skills they need for their constant improvement and performance.

## Data Availability

There are no data in this work.
